# Bacteria and macrophages in the tumor microenvironment

**DOI:** 10.3389/fmicb.2023.1115556

**Published:** 2023-02-07

**Authors:** Shiyao Xu, Yan Xiong, Beibei Fu, Dong Guo, Zhou Sha, Xiaoyuan Lin, Haibo Wu

**Affiliations:** School of Life Sciences, Chongqing University, Chongqing, China

**Keywords:** bacteria, cancer, tumor-associated macrophages, M1/M2 macrophage polarization, tumor microenvironment

## Abstract

Cancer and microbial infections are significant worldwide health challenges. Numerous studies have demonstrated that bacteria may contribute to the emergence of cancer. In this review, we assemble bacterial species discovered in various cancers to describe their variety and specificity. The relationship between bacteria and macrophages in cancer is also highlighted, and we look for ample proof to establish a biological basis for bacterial-induced macrophage polarization. Finally, we quickly go over the potential roles of metabolites, cytokines, and microRNAs in the regulation of the tumor microenvironment by bacterially activated macrophages. The complexity of bacteria and macrophages in cancer will be revealed as we gain a better understanding of their pathogenic mechanisms, which will lead to new therapeutic approaches for both inflammatory illnesses and cancer.

## 1. Bacterial diversity in different cancers

The immune system was traditionally thought to render tumors sterile. Thanks to technological advancements, numerous investigations in recent years have discovered that bacteria are prevalent in cancer. Many tumors’ early stages are challenging to recognize, and most malignancies have metastasized by the time of initial diagnosis. The classification of these bacteria may be able to provide us with some information. Testing of bacterial DNA has shown that each cancer type, including those not directly related to the external environment, has a different bacterial composition (Nejman et al., 2020). The goal of this section is to give a summary of the bacteria found in cancer tissue. To gather and compile the microorganisms, we specialize in nine common cancer types: colorectal, gastric, esophageal, pancreatic, gallbladder, lung, breast, cervical, and prostate (Table 1). Pathogenic bacteria and human commensal microorganisms frequently coexist in the enormous and complex microbial community that makes up the human gastrointestinal tract. The gastrointestinal microbiome has a significant impact on metabolic health and general health, and it is also the microbiome that has been studied the most in-depth and is used as a model to study host-microbiota interactions and disorders. In addition to being infrequently researched, the quantity of microbiota living in the other organs is substantially lower than that of the gut and stomach (Sepich-Poore et al., 2021). It’s uncertain how many and how diverse the bacteria are in cancer samples as compared to samples taken from healthy people. The quantity and diversity of bacteria are greater in breast tumor samples than in healthy, normal breast samples (Nejman et al., 2020). Breast cancer tissues of various grades and histological classifications and normal breast tissue differ greatly in terms of their bacterial composition (Nejman et al., 2020). Instead, the lung cancer tissue microbiome is less varied than the corresponding normal tissue microbiome (Peters et al., 2019). In reality, only one specific bacterial species, *Helicobacter pylori*, which is linked to gastric cancer designated by the World Health Organization as a class I carcinogen (Lunn et al., 2022), and research into other bacterial species’ potential roles as biomarkers in the majority of cancer types has not yet produced any conclusive findings (Scott et al., 2019). There is a large variety of microbial taxa with variable abundance but little overlap in studies of males with prostate cancer (Shrestha et al., 2018). Not only is there a microbiota within cancer, but crosstalk occurs in all organs of the body. The proliferation and composition of bacteria where not in direct contact with the outside world, to some extent, represent the bacteria that can be transferred from one organ to another. The microbiological makeup of the lungs is more similar to that of the oropharynx, and enteric organisms are the primary source of bacterial DNA in cancer patients’ pancreas tissue (Dickson and Huffnagle, 2015). Thanks to developments in polymerase chain reaction and metagenomics, we can now identify microbes more precisely. The researchers distinguish the microbiota of lung cancer tissue using different biological materials, such as bronchial or bronchoalveolar lavage fluid or sputum (Gomes et al., 2019), and the microbiota of prostate cancer using feces or urine microbiomes (Sfanos et al., 2008). But in every experiment, skin-associated germs could contaminate the reagents by transferring them from the personnel’s skin. Cancer-associated microbes in general are sometimes difficult to distinguish, and the study of particular bacteria in malignancies is still in its early stages.

**Table 1:** Summary of the cancer microbiome.

Cancer	Phylum	Genus	References
Colorectal cancer	*Bacteroidetes*	*Bacteroides*	Nejman et al. (2020), Wu et al. (2013), Bullman et al. (2017), Dejea et al. (2018), Kwong et al. (2018), Yachida et al. (2019)
Colorectal cancer	*Bacteroidetes*	*Prevotella*	Bullman et al. (2017), Dejea et al. (2018), Kwong et al. (2018), Wirbel et al. (2019)
Colorectal cancer	*Bacteroidetes*	*Porphyromonas*	Yachida et al. (2019), Wirbel et al. (2019), Thomas et al. (2019)
Colorectal cancer	*Firmicutes*	*Peptostreptococcus; Solobacterium*	Kwong et al. (2018), Wirbel et al. (2019), Thomas et al. (2019)
Colorectal cancer	*Firmicutes*	*Streptococcus*	Kwong et al. (2018), Yachida et al. (2019)
Colorectal cancer	*Firmicutes*	*Clostridium; Gemella*	Kwong et al. (2018), Wirbel et al. (2019))
Colorectal cancer	*Firmicutes*	*Lachnospiraceae*	Dejea et al. (2018), Yachida et al. (2019)
Colorectal cancer	*Firmicutes*	*Roseburia*	Coutzac et al. (2020)
Colorectal cancer	*Proteobacteria*	*Escherichia*	Arthur et al. (2012), Dejea et al. (2018), Thomas et al. (2019), Wilson et al. (2019), Pleguezuelos-Manzano et al. (2020)
Colorectal cancer	*Proteobacteria*	*Campylobacter*	He et al. (2019)
Colorectal cancer	*Actinobacteria*	*Bifidobacterium*	Shi et al. (2020)
Colorectal cancer	*Actinobacteria*	*Parvimonas*	Kwong et al. (2018), Yachida et al. (2019), Wirbel et al. (2019), Thomas et al. (2019)
Colorectal cancer	*Fusobacteria*	*Fusobacterium*	Wu et al. (2013), Bullman et al. (2017), Dejea et al. (2018), Kwong et al. (2018), Yachida et al. (2019), Wirbel et al. (2019), Thomas et al. (2019), Mima et al. (2016), Mima et al. (2015), Castellarin et al. (2012), Kostic et al. (2012), Kostic et al. (2013), Rubinstein et al. (2013), Eklof et al. (2017), Yu et al. (2017), Garrett (2019), Abed et al. (2020)
Stomach cancer	*Bacteroidetes*	*Alloprevotella*	Aviles-Jimenez et al. (2014), Roberts et al. (2002)
Stomach cancer	*Firmicutes*	*Parvimonas*	Aviles-Jimenez et al. (2014), Roberts et al. (2002), Coker et al. (2018), Nagano et al. (2019), Baghban and Gupta (2016), Kim et al. (2010)
Stomach cancer	*Firmicutes*	*Dialister*	Aviles-Jimenez et al. (2014), Roberts et al. (2002), Coker et al. (2018), Nagano et al. (2019), Wang L. L. et al. (2014)
Stomach cancer	*Firmicutes*	*Streptococcus*	Coker et al. (2018), Nagano et al. (2019), Hsieh et al. (2018), Li et al. (2016), Zhao et al. (2015)
Stomach cancer	*Firmicutes*	*Slackia*	Aviles-Jimenez et al. (2014), Roberts et al. (2002), Coker et al. (2018), Nagano et al. (2019), Schulz et al. (2019), Contreras et al. (2000)
Stomach cancer	*Firmicutes*	*Lactobacillus*	Aviles-Jimenez et al. (2014), Hsieh et al. (2018)
Stomach cancer	*Firmicutes*	*Clostridium*	Hsieh et al. (2018), Salazar et al. (2013)
Stomach cancer	*Firmicutes*	*Staphylococcus*	Roberts et al. (2002), Weng et al. (2019)
Stomach cancer	*Firmicutes*	*Veillonella*	Dias-Jacome et al. (2016)
Stomach cancer	*Proteobacteria*	*Helicobacter*	Hsieh et al. (2018), Suzuki et al. (2009)
Stomach cancer	*Proteobacteria*	*Neisseria*	Aviles-Jimenez et al. (2014), Li et al. (2016)
Stomach cancer	*Proteobacteria*	*Sphingobium*	Dias-Jacome et al. (2016)
Stomach cancer	*Proteobacteria*	*Escherichia; Burkholderia*	Li et al. (2016)
Stomach cancer	*Fusobacteria*	*Fusobacterium*	Hsieh et al. (2018)
Esophageal cancer	*Firmicutes*	*Lactobacillus; Streptococcus*	Elliott et al. (2017)
Esophageal cancer	*Fusobacteria*	*Fusobacterium*	Yamamura et al. (2016)
Pancreatic cancer	*Bacteroidetes*	*Porphyromonas*	Poore et al. (2020), Riquelme et al. (2019), Pushalkar et al. (2018), Geller et al. (2017)
Pancreatic cancer	*Firmicutes*	*Streptococcus; Granulicatella*	Farrell et al. (2012)
Pancreatic cancer	*Proteobacteria*	*Pseudoxanthomonas*	Riquelme et al. (2019)
Pancreatic cancer	*Proteobacteria*	*Neisseria*	Farrell et al. (2012)
Pancreatic cancer	*Actinobacteria*	*Saccharopolyspora; Streptomyces*	Riquelme et al. (2019), Geller et al. (2017)
Gallbladder cancer	*Bacteroidetes*	*Bacteroidaceae; Prevotellaceae; Porphyromonadaceae*	Molinero et al. (2019)
Gallbladder cancer	*Firmicutes*	*Veillonellaceae*	Molinero et al. (2019)
Gallbladder cancer	*Proteobacteria*	*Salmonella*	Dutta et al. (2000), Nagaraja and Eslick (2014), Nath et al. (2008), Nath et al. (2010)
Gallbladder cancer	*Proteobacteria*	*Helicobacter*	de Martel et al. (2009), Pradhan and Dali (2004), Murata et al. (2004)
Gallbladder cancer	*Proteobacteria*	*Escherichia*	Tsuchiya et al. (2018)
Gallbladder cancer	*Proteobacteria*	*Enterobacteriaceae*	Tsuchiya et al. (2018)
Gallbladder cancer	*Fusobacteria*	*Fusobacterium*	Tsuchiya et al. (2018)
Lung cancer	*Bacteroidetes*	*Prevotella*	Tsay et al. (2018), Dickson et al. (2016), Tsay et al. (2021)
Lung cancer	*Bacteroidetes*	*Capnocytophaga*	Liu et al. (2018), Yan et al. (2015)
Lung cancer	*Firmicutes*	*Streptococcus*	Tsay et al. (2018), Dickson et al. (2016), Tsay et al. (2021), Liu et al. (2018), Apostolou et al. (2011), Laroumagne et al. (2013), Hosgood et al. (2014), Cameron et al. (2017)
Lung cancer	*Firmicutes*	*Veillonella*	Tsay et al. (2018), Dickson et al. (2016), Tsay et al. (2021), Yan et al. (2015), Lee et al. (2016)
Lung cancer	*Firmicutes*	*Staphylococcus*	Dickson et al. (2016), Laroumagne et al. (2013)
Lung cancer	*Firmicutes*	*Lactobacillus*	Tsay et al. (2021), Jin et al. (2019)
Lung cancer	*Firmicutes*	*Gemella*	Tsay et al. (2021)
Lung cancer	*Firmicutes*	*Selenomonas*	Yan et al. (2015)
Lung cancer	*Firmicutes*	*Enterococcus*	Cameron et al. (2017)
Lung cancer	*Firmicutes*	*Megasphaera*	Lee et al. (2016)
Lung cancer	*Proteobacteria*	*Enterobacter*	Dickson and Huffnagle (2015), Gomes et al. (2019), Laroumagne et al. (2013), Cameron et al. (2017)
Lung cancer	*Proteobacteria*	*Acinetobacter*	Gomes et al. (2019), Cameron et al. (2017)
Lung cancer	*Proteobacteria*	*Haemophilus*	Tsay et al. (2021), Laroumagne et al. (2013)
Lung cancer	*Proteobacteria*	*Burkholderia*	Dickson et al. (2016), Tsay et al. (2021)
Lung cancer	*Proteobacteria*	*Moraxella*	Tsay et al. (2021)
Lung cancer	*Proteobacteria*	*Neisseria*	Yan et al. (2015)
Lung cancer	*Proteobacteria*	*Noviherbaspirillum; Aggregatibacter*	Jin et al. (2019)
Lung cancer	*Proteobacteria*	*Brevundimonas*	Dickson and Huffnagle (2015), Gomes et al. (2019)
Lung cancer	*Proteobacteria*	*Acidovorax*	Greathouse et al. (2018)
Lung cancer	*Proteobacteria*	*Morganella; Escherichia*	Le Noci et al. (2018)
Lung cancer	*Proteobacteria*	*Legionella*	Yu et al. (2016)
Lung cancer	*Actinobacteria*	*Rothia*	Tsay et al. (2018), Tsay et al. (2021)
Lung cancer	*Actinobacteria*	*Propionibacterium*	Dickson and Huffnagle (2015), Gomes et al. (2019)
Lung cancer	*Fusobacteria*	*Fusobacterium*	Tsay et al. (2021)
Lung cancer	*Deinococcus-Thermus*	*Thermus*	Yu et al. (2016)
Lung cancer	*Verrucomicrobia*	*Akkermansia*	Derosa et al. (2018), Routy et al. (2018)
Breast cancer	*Firmicutes*	*Bacillus; Staphylococcus*	Urbaniak et al. (2016)
Breast cancer	*Proteobacteria*	*Enterococcus*	Urbaniak et al. (2016)
Breast cancer	*Fusobacteria*	*Fusobacterium*	Parhi et al. (2020)
Cervical cancer	*Bacteroidetes*	*Prevotella*	So et al. (2020), Onderdonk et al. (2016)
Cervical cancer	*Firmicutes*	*Lactobacillus*	Poore et al. (2020), Pearce et al. (2014)
Cervical cancer	*Firmicutes*	*Dialister; Finegoldia Magna; Peptoniphilus*	So et al. (2020)
Cervical cancer	*Firmicutes*	*Parvimonas; Peptostreptococcus; Anaerococcus*	Onderdonk et al. (2016)
Cervical cancer	*Firmicutes*	*Clostridium*	Donders et al. (2017)
Cervical cancer	*Firmicutes*	*Streptococcus*	Donders et al. (2017), Liu et al. (2020)
Cervical cancer	*Firmicutes*	*Megasphaera*	Onderdonk et al. (2016), Fredricks et al. (2005)
Cervical cancer	*Proteobacteria*	*Hydrogenophilus; Burkholderia*	Zhou Y. et al. (2019)
Cervical cancer	*Actinobacteria*	*Atopobium*	So et al. (2020), Onderdonk et al. (2016), Fredricks et al. (2005), Gondwe et al. (2020)
Cervical cancer	*Actinobacteria*	*Gardnerella*	So et al. (2020), Onderdonk et al. (2016), Pearce et al. (2014), Zhou Y. et al. (2019)
Cervical cancer	*Actinobacteria*	*Eggerthella*	Fredricks et al. (2005)
Cervical cancer	*Actinobacteria*	*Bifidobacterium*	Zhou Y. et al. (2019)
Cervical cancer	*Fusobacteria*	*Sneathia*	Onderdonk et al. (2016), Zhou Y. et al. (2019), Gondwe et al. (2020), Lee et al. (2013), Mitra et al. (2015), Audirac-Chalifour et al. (2016), Di Paola et al. (2017), Laniewski et al. (2018)
Cervical cancer	*Fusobacteria*	*leptotrichia*	Fredricks et al. (2005)
Cervical cancer	*Fusobacteria*	*Fusobacterium*	Zhou Y. et al. (2019)
Prostate cancer	*Bacteroidetes*	*Bacteroides*	Keay et al. (1999), Golombos et al. (2018), Liss et al. (2018), Alanee et al. (2019)
Prostate cancer	*Firmicutes*	*Staphylococcus*	Shrestha et al. (2018), Cavarretta et al. (2017)
Prostate cancer	*Firmicutes*	*Streptococcus*	Shrestha et al. (2018), Liss et al. (2018)
Prostate cancer	*Firmicutes*	*Faecalibacterium*	Miquel et al. (2013), Sokol et al. (2008)
Prostate cancer	*Firmicutes*	*Clostridium*	Ridlon et al. (2013)
Prostate cancer	*Proteobacteria*	*Escherichia*	Keay et al. (1999), Leskinen et al. (2003)
Prostate Cancer	*Proteobacteria*	*Proteus; Aeromonas*	Leskinen et al. (2003)
Prostate cancer	*Proteobacteria*	*Campylobacter*	Lara-Tejero and Galan (2000)
Prostate cancer	*Actinobacteria*	*Propionibacterium*	Sfanos et al. (2008), Cavarretta et al. (2017), Cohen et al. (2005)
Prostate cancer	*Actinobacteria*	*Corynebacterium*	Shrestha et al. (2018), Daisley et al. (2020)
Prostate cancer	*Verrucomicrobia*	*Akkermansiaceae*	Daisley et al. (2020)

The species of bacteria have been reported in studies that present in cancer.

## 2. Macrophages in the tumor microenvironment

Macrophages are multipurpose immune cells that perform a variety of tasks, such as regulating tissue homeostasis, protecting against infections, and accelerating wound healing (Wynn and Vannella, 2016). Macrophages are found in peripheral organs because these immunological sentinel cells are crucial in keeping an eye out for invasive infections in the surrounding tissue (Sfanos et al., 2008). When a host is infected by a pathogen, monocytes are drawn to the invasion sites and cytokines are released, which prompt additional immune responses from other immune cells. Indeed, bacteria and their metabolites have recently been shown to affect macrophages and tumor microenvironments. Numerous disorders, including cancer and infections for which there is yet no clear direct link, are affected by macrophage activation. Here, we discuss macrophages from three angles: their origin, their activation indicators, and the bacterial collection that causes them to become polarized. We just briefly touch on the preceding two aspects, because they have recently been discussed (Murray et al., 2014; Wynn and Vannella, 2016; Shapouri-Moghaddam et al., 2018; Christofides et al., 2022). Instead, we focus on the results of macrophage polarization caused by certain bacteria.

### 2.1. The source of macrophages

All tissues have macrophages, a kind of leukocyte that is divided into various subpopulations according to where it is found and how it functions. Macrophages come from two different origins. On the other hand, tissue-resident macrophages derived from erythro-myeloid progenitors in the yolk sac and fetal liver, or monocyte–macrophage DC progenitors in the bone marrow (Cassetta and Pollard, 2020). Peripheral blood monocytes, which are drawn to tissues by chemokines, can also develop into tissue-resident macrophages (Long et al., 2019). One of the numerous and varied cell groups that make up the tumor microenvironment and can affect tumor formation is the tumor-associated macrophages that populate the tumor tissue (Vitale et al., 2019). It is firmly established that TAMs influence tumor development, immunological control, tumor angiogenesis, and metastasis in the tumor microenvironment (Lin et al., 2019). Although the precise timing and process of this remain unknown, the bulk of TAMs are typically produced from blood monocytes, and tumor monocytes recruited *via* chemokines like CCL2 enter the tumor to develop into TAMs (Mantovani et al., 2008). Additionally, macrophages in metastatic tumors often referred to as metastasis-associated macrophages (MAMs), have different phenotypes and roles from those in primary tumors. TAMs states in patients have predictive relevance, according to some research, as their abundance correlates with various clinical outcomes (Pittet et al., 2022).

### 2.2. The polarization and markers of macrophages

Macrophage polarization is a biological process that eventually displays a certain phenotype after functionally responding to microenvironmental signals found in particular tissues. M1/M2 is acknowledged as the most straightforward word to describe macrophage phenotypes, based on the types of activation signals, such as immunological signals, tumor metabolism signals, and cell death signals. The M1 macrophage, a type of classical activation macrophage, plays a crucial role in anti-tumor immunity as well as mediating the host’s defense against a variety of bacteria, protozoa, and viruses. It is also involved in several chronic inflammatory and autoimmune illnesses (Murray and Wynn, 2011). Conversely, the M2 macrophage is an alternatively activated macrophage that devours fragmented and apoptotic cells and has anti-inflammatory and pro-angiogenic activity to control wound healing (Murray and Wynn, 2011). Depending on the activating stimuli received, M2 macrophages have been further divided into M2a, M2b, M2c, and M2d (Shapouri-Moghaddam et al., 2018). TAMs are a polarized novel subset of the M2 macrophage population, which was named M2d in recent studies (Mantovani et al., 2002; Duluc et al., 2007). M1-polarized cells produce ROS and NO more effectively, as well as pro-inflammatory cytokines like TNF-α, IL-1, and IL-6, as well as chemokines like CXCL8, CCL2, CXCL9, and CXCL10. M2-polarized cells express the mannose receptor, which triggers the production of chemokines including CCL17, CCL18, CCL22, and CCL24. They also produce anti-inflammatory cytokines like IL-10 and TGF-β. The expression of macrophage activation indicators, as well as cytokines, chemokines, and other secreted mediators, have all been thoroughly discussed in these papers (Mantovani et al., 2004; Murray et al., 2014; Shapouri-Moghaddam et al., 2018). TAM subpopulations are also first categorized as M1 and M2 macrophages based on the expression of certain markers, with functions assumed to be anti-tumor/anti-inflammation and pro-tumor/pro-inflammation development, respectively. It is usually determined that the majority of TAMs isolated from primary and metastatic cancers exhibit a suppressive M2-like phenotype (Murray and Wynn, 2011). However, because numerous subsets of TAMs display the indicators of both M1-and M2-polarization signatures, this simple nomenclature is unable to discriminate between the varied phenotypes of TAMs (Laviron and Boissonnas, 2019). TAMs are not precisely divided into the M1 and M2 phenotypes *in vivo*. How to select reliable biomarkers to classify TAMs in the marker database remains the main issue of research nowadays.

### 2.3. Macrophage polarization by bacteria

**Figure 1:**
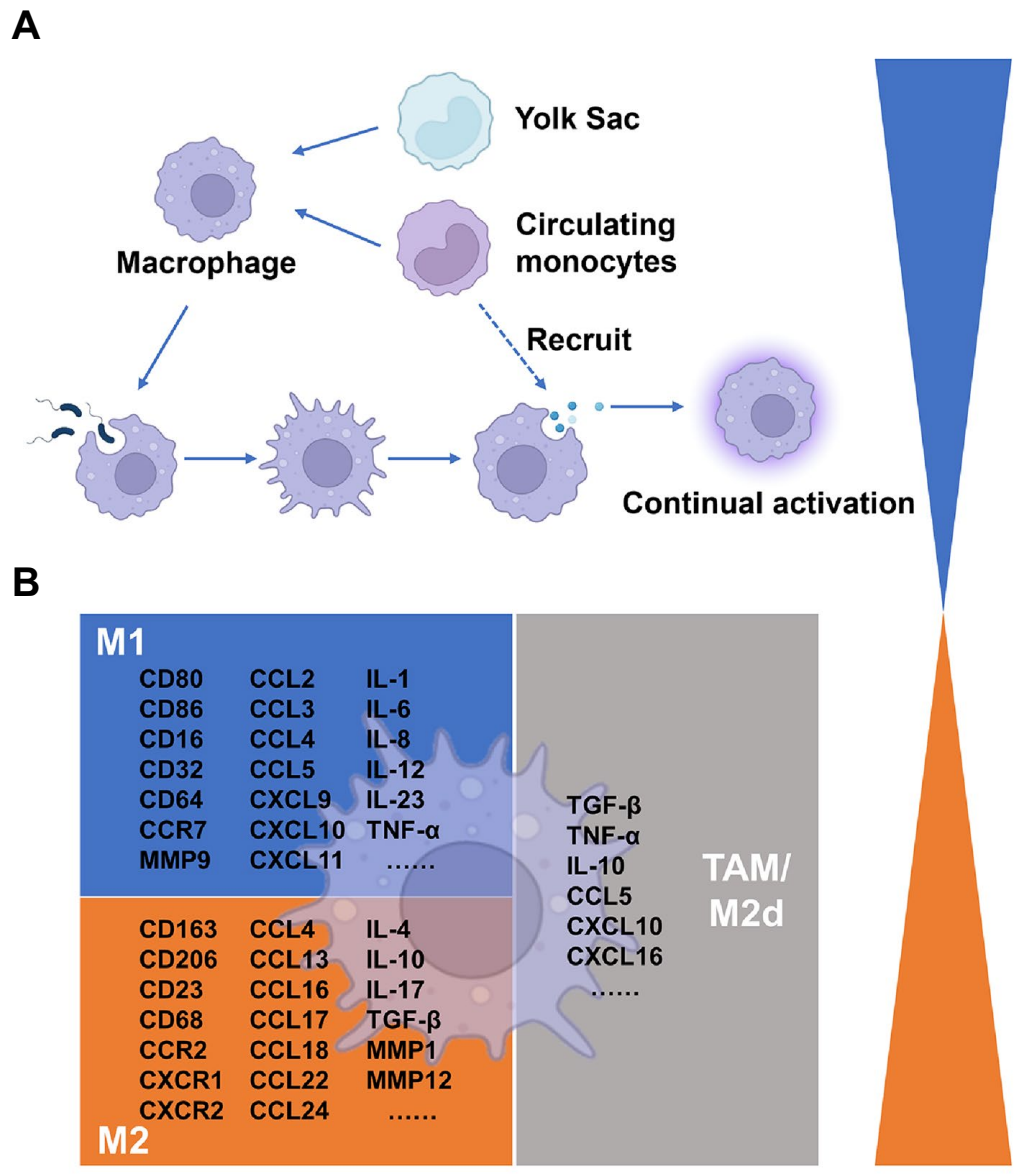
Summary of macrophages in the tumor microenvironment. **(A)** Origins of Macrophages. **(B)** Cytokines associated with macrophage differentiation to the classically and alternatively activated subsets.

In response to cues from the immediate milieu, macrophages can change from one functional phenotype to another. Particular signaling sources like pathogenic sources might cause phenotypic flipping in macrophage populations and promote the development of tumors. Although most microorganisms are phagocytosed and killed by macrophages, some bacteria live in macrophages as opportunistic residents and utilize them for replication (Geller et al., 2017). M1 macrophages are mediated by microbial stimuli, including intracellular bacteria, to support cytotoxic activity and infection resistance. To thrive in the microenvironment, some bacteria can increase M2 polarization or interfere with M1 polarization. To avoid cytotoxic effects and circumvent the cellular immune response, microbes like Mycobacterium tuberculosis may mediate M2-polarized macrophages (Kaufmann, 2016). Table 2 lists the phenotypes of macrophages in response to bacterial pathogens in the context of oncology. Our understanding of functional markers may be too simplistic, while the use of complex markers may be confusing for researchers outside of immunology. All things considered, we continue to define pro-tumor/pro-inflammatory macrophages as M1 and anti-tumor/anti-inflammation macrophages as M2. However, it should not be forgotten that a particular live scene is unlikely to fall exactly into the combinations in Table 2, and a deeper study is needed to obtain further information and standardization (Figure 1).

**Table 2:** An aggregate list of bacterial-driven macrophage polarization that has been studied is currently available.

*Porphyromonas*	*Porphyromonas gingivalis*	M1	Li et al. (2022)
*Coxiella*	*Coxiella burnetii*	M1	Abnave et al. (2017), Zarza et al. (2021)
*Escherichia*	*Escherichia coli*	M1	Liang et al. (2005), Pinheiro da Silva et al. (2007), Christoffersen et al. (2014)
*Yersinia*	*Yersinia pestis*	M1	Bi et al. (2012)
*Legionella*	*Legionella pneumophila*	M1	Kusaka et al. (2018)
*Vibrio*	*Vibrio cholerae*	M1	Khan et al. (2015)
*Shigella*	*Shigella dysenteriae*	M1	Biswas et al. (2007), Pore et al. (2010)
*Streptococcus*	*Streptococcus pyogenes*	M1	Kadioglu and Andrew (2004), Goldmann et al. (2007)
*Streptococcus*	*Streptococcus Gordonii*	M1	Croft et al. (2018)
*Lacticaseibacillus*	*Lactobacillus rhamnosus GG*	M1	Wang et al. (2020), Duan et al. (2021)
*Bacillus*	*Bacillus amyloliquefaciens*	M1	Fu et al. (2019)
*Mycobacterium*	*Mycobacterium ulcerans*	M1	Kiszewski et al. (2006)
*Mycobacterium*	*Mycobacterium avium*	M1	Murphy et al. (2006)
*Mycobacterium*	*Mycobacterium tuberculosis*	M1/M2	Ehrt et al. (2001), Chacon-Salinas et al. (2005), Huang et al. (2015), Sha et al. (2021), Lopes et al. (2016), Zhang et al. (2020a)
*Mycobacterium*	*Mycobacterium leprae*	M1/M2	Fallows et al. (2016)
*Salmonella*	*Salmonella typhimurium*	M1/M2	Luo et al. (2016), Monack et al. (1996), Bost and Clements (1997), Stapels et al. (2018)
*Fusobacterium*	*Fusobacterium nucleatum*	M1/M2	Wu et al. (2019), Xu et al. (2021), Chen et al. (2018)
*Streptococcus*	*Streptococcus pneumonia*	M2/M1	Yerneni et al. (2021), Smith et al. (2007)
*Staphylococcus*	*Staphylococcus aureus*	M2/M1	Peng et al. (2017), Pidwill et al. (2020), Tuohy et al. (2020)
*Listeria*	*Listeria monocytogenes*	M2/M1	Lizotte et al. (2014), Shaughnessy and Swanson (2007)
*Tropheryma*	*Tropheryma whipplei*	M2	Desnues et al. (2005)
*Cutibacterium*	*Propionibacterium acnes*	M2	Li et al. (2021)
*Bifidobacterium*	*Bifidobacterium pseudocatenulatum*	M2	Sohn et al. (2015)
*Bacillus*	*Bacillus subtilis*	M2	Paynich et al. (2017)
*Lacticaseibacillus*	*Lactobacillus paracasei KW3110*	M2	Yoshikawa et al. (2021), Moratalla et al. (2016)
*Enterococcus*	*Enterococcus faecalis*	M2	Polak et al. (2021)
*Brucella*	*Brucella abortus*	M2	Fernandes et al. (1996), Wang et al. (2022), Dornand et al. (2002), Glowacka et al. (2018), Ma et al. (2020)
*Brucella*	*Brucella melitensis*	M2	Wang et al. (2022)
*Helicobacter*	*Helicobacter pylori*	M2	Wang et al. (2017)

## 3. Molecular mechanisms involved in bacterial-driven macrophage polarization

Since the microbiota has existed in the gut since human birth, the immune system and bacteria may have always been connected. Different bacterial species use both common and distinctive intrinsic mechanisms to either support or kill cancer. When it comes to host-pathogen interactions, living bacteria or bacterial components typically trigger innate immune cell reactions and cause immune cells, such as monocytes and macrophages, to migrate to tumors. Several bacteria are known to be connected to cancer, such as *Helicobacter pylori* and *Salmonella typhi* that have been shown to affect tumor growth (Lax and Thomas, 2002). However, the method by which bacteria in tumors interact with macrophages to select for the M1/M2 activation pathway is rarely discussed. In this part, we examine the molecular processes by which typical bacteria influence the polarization of macrophages in tumors, focusing on some of the well-known strains, such as *Fusobacterium nucleatum*, *Helicobacter pylori*, and *Propionibacterium acnes*.

### 3.1. Bacteria significantly linked to cancer

#### 3.1.1. Fusobacterium nucleatum

A member of the bacterial genus that may cause cancer is called *Fusobacterium nucleatum*, a Gram-negative anaerobic bacterium. *Fusobacterium nucleatum* has been discovered as a periodontal pathogen and has been preferentially isolated from the oral cavity. Additionally, it has been extensively addressed how *F. nucleatum* and colorectal cancer are related (Hashemi Goradel et al., 2019). The Toll-like receptors recognize the molecular characteristics of pathogens, and each TLR elicits a different cellular response to the pathogen. In the microenvironment of colorectal tumors, *F. nucleatum* has been shown to enhance macrophage M2 polarization through a TLR4-dependent mechanism. Infection with *F. nucleatum* may also activate the IL-6/p-STAT3/c-MYC signaling pathway in macrophages in a TLR4-dependent manner (Chen et al., 2018). The study further illustrates that *F. nucleatum* promotes M2 macrophage polarization through activation of the TLR4/NF-κB/S100A9 cascade (Hu et al., 2021). The transcriptional stimulation of downstream NF-κB and STAT3, which can activate the survival pathway of tumor cells, is one of the main functions of TLR signaling. *Fusobacterium nucleatum* has been demonstrated to promote the growth of colorectal cancer *via* stimulating TLR4 signaling to MyD88, which then triggers the nuclear factor NF-κB and miR21 production (Yang et al., 2017). *Fusobacterium nucleatum* regulates miR-1,322/CCL20 through the NF-κB signaling pathway in colorectal cancer cells ultimately inducing macrophage M2 polarization (Xu et al., 2021). By releasing bioactive chemicals, bacteria can impact the host or nearby cells. A potential new marker for colorectal cancer is AI-2 in the gut microbiota (Li et al., 2019). Interestingly, AI-2 of *F. nucleatum* can promote macrophage M1 polarization *via* TNFSF9/IL-1β signaling (Wu et al., 2019).

#### 3.1.2. Helicobacter pylori

*Helicobacter pylori* is a Gram-negative bacterium that when infected can cause chronic gastritis and subsequently increase the risk of developing gastric tumors in infected patients. M2-polarized macrophages identified by the CD163 molecule were substantially expressed in gastric cancer and lowly expressed in marginal tissues, implying that macrophage polarization is intimately related to gastric cancer (Zhu et al., 2020). Macrophages detect the presence of pathogen-associated molecular patterns from *H. pylori* using PRRs such as TLRs and NLRs. Inflammation caused by *H. pylori* infection is associated with the expression of TLR4 and TLR9. TLR9 is found in the intracellular compartment and can recognize nucleic acids from bacteria. TLR4 plays a major role in the inflammation of the superficial gastric lining, whereas TLR9 plays a major role in the inflammation of gastric cancer (Wang T. R. et al., 2014). Bacterial peptidoglycan particles are detectable by NOD1. NOD1 collaborates with TLRs to detect bacteria and mediate the production of inflammatory factors. Loss of NOD1 accelerates stomach carcinogenesis in a mouse model. The wild-type phenotype of macrophages rapidly changed from M2 to M1 after the *H. pylori* infection. While wild-type macrophages convert to a mixed M1-M2 phenotype after infection with *H. pylori*, NOD1-deficient macrophages exhibit a more pronounced M2 phenotype (Suarez et al., 2019). Another study found that the deletion of MMP7 boosted M1 macrophage polarization and raised the risk of gastric cancer brought on by *H. pylori* (Krakowiak et al., 2015). Certain miRNAs may play a role in the bacterial infection’s ability to persist. *Helicobacter pylori* can upregulate miRNAs targeting CIITA, thereby suppressing HLAII expression on macrophages which plays a key role in the presentation of antigens to T lymphocytes (Codolo et al., 2019; Coletta et al., 2021). After an *H. pylori* infection, macrophages regulate the release of proinflammatory cytokines *via* the increased expression of miR-155 (Yao et al., 2015).

### 3.2. Bacteria significantly linked to infection

#### 3.2.1. Propionibacterium acnes

*Propionibacterium acnes* is a Gram-positive bacterium known as a cutaneous commensal. However, it can also manifest as an opportunistic pathogen, which gives the impression of intrusion. Through TLR4/PI3K/Akt signaling, *P. acnes* encourages M2 macrophage polarization in gastric cancer (Li et al., 2021). Regardless of the existence of malignancy, *P. acnes* infection can be found in the macrophages and epithelial cells of the prostate gland. However, persistent inflammation is linked to *P. acnes*-positive macrophage populations and is most likely a factor in the development of cancer. Both TLR4 and TLR2 are capable of identifying lipids and the LPS that Gram-negative bacteria generate. Interestingly, Kim et al. showed that *P. acnes* triggered an inflammatory response in macrophages by the activation of TLR2 while the TLR ligand may be the peptidoglycan (Kim et al., 2002). Due to the late discovery of the pathogenicity of *P. acnes*, little is known about this bacterium.

#### 3.2.2. Staphylococcus aureus

*Staphylococcus aureus* is the leading causative agent in pneumonia and is initially cleared from the bloodstream by liver macrophages also called Kupffer cells. These infected cells will spread intracellular *S. aureus* throughout the body if they are unable to kill it, leading to disseminated infection. The virulence regulation of *S. aureus* is more sophisticated than that of many other bacterial pathogens. When combined with a strong Arg-1 induction, *S. aureus* biofilms can reduce iNOS expression and drive M2 macrophage polarization (Thurlow et al., 2011). In extramammary Paget S disease, *S. aureus* may be exacerbated by IL-17 and M2 macrophage polarization (Tuohy et al., 2020; Sakamoto et al., 2021). TGF-β levels were lower and inflammatory cytokines were more prominent in macrophages exposed to *S. aureus* in co-culture with osteosarcoma (Tuohy et al., 2020). The majority of the time, significant expression of conventional HDAC enzymes are linked to cancer, and it frequently indicates advanced disease and a poor prognosis for the patient. Interestingly, *S. aureus*-derived lactate inhibits the negative regulator HDAC11 to augment leukocyte IL-10 production in an HDAC6-dependent manner in the mouse prosthetic joint infection model (Heim et al., 2020). IL-10 expression correlated with the expression of HDAC6 and HDAC11 was also reported in *M. tuberculosis* infection (Wang et al., 2018).

### 3.3. Engineered bacteria

#### 3.3.1. Bacillus Calmette-Guérin

Natural bacteria have been modified to acquire therapeutic functions as a result of the development of bioengineering technology. Bacillus Calmette-Guérin, a vaccine against tuberculosis, contains live-attenuated and non-toxic *M. tuberculosis*. The most advanced immunotherapy now available for non-muscle-invasive bladder cancer is BCG (Seow et al., 2010). To prevent the growth of malignancies, BCG instructs monocyte precursor cells to differentiate into functioning mature macrophages (Italiani and Boraschi, 2014). The pathogen BCG activates the MyD88 signaling pathway downstream of the cell surface TLRs, which in turn activates NF-κB and encourages cytokine transcription (de Queiroz et al., 2021). Through the TLR2/TLR4/IRF5 pathway, TRIM59 expression is elevated in BCG-activated macrophages (Jin et al., 2017). TRIM59 is a membrane protein expressed on macrophages that can increase the M1-polarized macrophages inside the tumor (Tian et al., 2019). In addition, BCG inhibits cervical carcinoma progression by promoting M1 macrophage polarization and inhibiting the pro-tumor activation of M2 macrophages *via* the Rb/E2F1 signaling pathway in Hela cells (Liu et al., 2021).

#### 3.3.2. Salmonella

**Figure 2:**
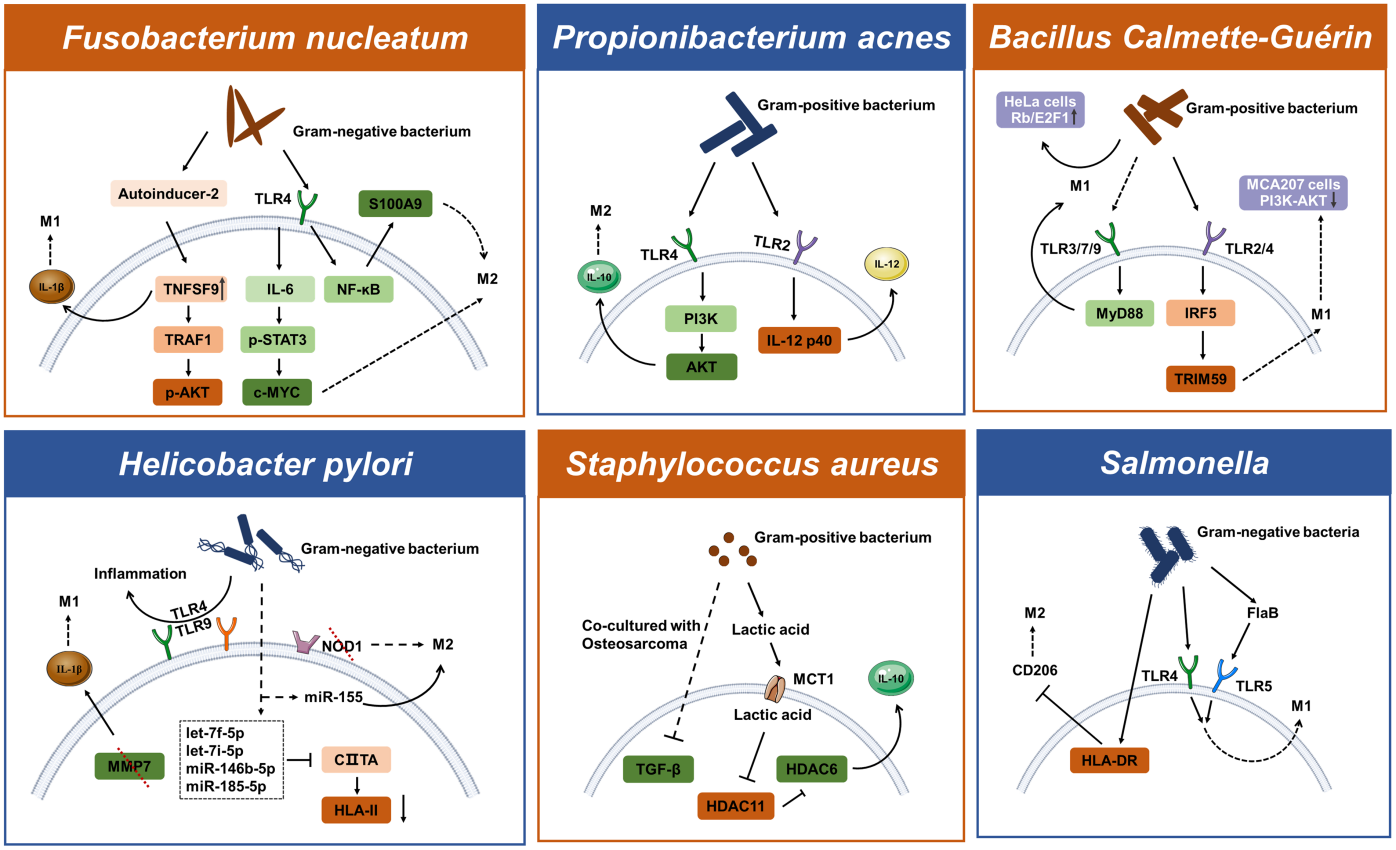
Schematic representation of mechanisms of bacteria-induced macrophage polarization.

*Salmonella* species are facultative intracellular pathogenic bacteria that can invade and proliferate in macrophages and dendritic cells. In *Salmonella*-infected macrophages, the fatty acid regulator PPARδ is increased and may be linked to M2-polarized macrophages (Eisele et al., 2013) and the PI3K/Akt pathway in gallbladder cancer can facilitate migration and invasion due to CCL18 produced by M2 macrophages (Zhou Z. et al., 2019). While some studies suggest that engineered *Salmonella* bacteria help tumor-associated macrophage polarization (see Figure 2) to achieve enhanced antitumor immune response *in vivo*. The anti-tumor effect of engineered *Salmonella* also appears to induce infiltration of abundant immune cells through TLR4 signaling. Molecular mechanisms suggest that this may be due to the presence of LPS in the outer membrane of Gram-negative bacteria thereby activating the TLR4/MyD88 pathway that mediated CCL2 production (Akhter et al., 2018). Some of the attenuated *Salmonella* strains and their derivatives used as drug carriers have also been tested in early clinical trials. A newly engineered *Salmonella typhimurium* strain called YB1 was reported to induce enhanced HLA-DR expression and reduced CD206 expression, and to remodel macrophages from the M2-to M1-polarized (Yang et al., 2018). Additionally, heterologous flagellin from the bacterial pathogen *Salmonella* activates the TLR5 pathway and changes tumor-infiltrating macrophages into M1-polarized macrophages (Chen et al., 2021). Another engineered bacteria that secrete FlaB *via* dual pathways, TLR4 and TLR5, also leads to M1-polarized macrophages (Zheng J. H. et al., 2017).

## 4. Effect of activated macrophages on the tumor microenvironment

While studies targeting the direct relationship between cancer and microbiome are quite limited at present, there have been some interesting studies demonstrating that certain pathogenic processes, such as altered metabolic states and chronic inflammation, display commonality across cancers (Trinchieri, 2012; Andrejeva and Rathmell, 2017). Microbiota participates in shaping an immune-tolerant environment through the recruitment and activation of macrophages and is characterized by the accumulation of pro-inflammatory factors including metabolic intermediates and effectors. These pro-inflammatory factors aid in the development of cancer by promoting angiogenesis, chemoresistance, immune cell suppression, tumor invasion, and metastasis (Coussens and Werb, 2002).

### 4.1. Dynamic changes in macrophage metabolism

The components of pathogenic organisms, such as LPS are commonly used tools to activate macrophages. LPS stimulation and TLR activation induce a series of biochemical metabolic alterations in macrophages. Metabolomic analysis of LPS-activated macrophages shows downregulation of TCA cycle intermediates and upregulation of aerobic glycolysis, which correlates directly with the expression profiles of altered metabolites (Tannahill et al., 2013; Lauterbach et al., 2019). The TCA cycle is a fragmented process resulting in the secretion of large volumes of metabolites such as lactate and succinate. The production of lactate in LPS-activated macrophages is enhanced and it inhibits the motility of activated T cells *in vitro* (Haas et al., 2015) and the cytotoxic activity of CD8 + CTLs (Fischer et al., 2007). By activating HIF-1α and MAPK, abundant lactate causes macrophages to produce VEGF and ARG1. Both VEGF and ARG1 promote tumor progressions by inducing angiogenesis and arginase depletion. Succinate is a pro-inflammatory metabolite that inhibits prolyl hydroxylase activity and increases the production of ROS, which stabilizes HIF-1α (Liu et al., 2017). Several genes that promote tumor growth, including MMP9, are also activated by HIF (Zhang et al., 2015). Furthermore, considering that the HIF protein amount is under the control of iron-dependent prolyl hydroxylases (Bruick and McKnight, 2001) and Lcn-2 promotes downstream target gene activation (Bolignano et al., 2010), iron uptake is another mechanism behind the pro-tumorigenic activity of polarized macrophages. Iron is known to regulate the expression of several genes at the transcriptional level, most prominently *via* the generation of reactive oxygen species and their effects on the activity of NF-κB and other transcription factors (Templeton and Liu, 2003). Iron stimulates cell production of hydroxyl radicals through the overexpression of SOX9, which regulate tumor aggressiveness (Chanvorachote and Luanpitpong, 2016). Under infectious or inflammatory conditions, macrophages increase iron absorption while promoting inflammation (Jung et al., 2015). While macrophages infected with extracellular *E. coli* K88 reserve iron by elevating hepcidin transcription and increasing iron storage in cells, macrophages infected with intracellular *S. typhimurium* decrease free iron ions for intracellular bacterial proliferation and utilization (Gan et al., 2019). Reduced intracellular iron levels in macrophages prevent inflammatory cytokines like IL-6 and TNF-α from being translated (Wang et al., 2008). A key mechanism for pathogens to perturb the biochemical metabolism to promote their survival in macrophages is lipid metabolism. Lipid metabolism is a crucial way by which infections disrupt metabolic metabolism to aid in their survival in macrophages. Lipid droplets, which are now acknowledged as a well-established characteristic of many tumors, accumulate excessive amounts of lipids and cholesterol (Beloribi-Djefaflia et al., 2016). By changing the metabolism of host cells, *M. tuberculosis* encourages the production of macrophages with lipid bodies (Russell et al., 2009). *Helicobacter pylori* engagement of the intracellular NOD1 leads to the activation of NF-κB, which results in the up-regulation of COX-2 (Chang et al., 2004), and COX-2 plays a key role in the synthesis of lipid inflammatory mediators such as prostaglandins from arachidonic acid. In addition, microbial stimulation triggers the expression of SREBP-1a (Im et al., 2011) and the synthesis of phosphatidylcholine (Sanchez-Lopez et al., 2019), which is linked to the production of IL-1β and IL-18 (Oishi et al., 2017). Increases in dephosphorylation of SHP1 caused by higher levels of oxidative stress from fatty acid oxidation are correlated with tumor progression and involve a variety of immune cell types (Myers et al., 2020; Su et al., 2020).

### 4.2. Populations and expression of regulatory inflammatory factors in macrophage

In the context of immunity, activated macrophages undergo metabolic adjustments and modify the production of cytokines at the epigenetic, transcriptional, and post-translational levels in response to bacterial sensing. Furthermore, the release of increased concentrations of intermediates and effectors frequently controls the tumor immune microenvironment and aids in the development of tumors. TAMs attract naïve and Th2 lymphocytes and cause inefficient immunological reactions by secreting CCL17, CCL18, and CCL22 (Erreni et al., 2011). Additionally, through producing CCL18, which binds to PITPNM3 on the cancer cell membrane, TAMs in breast cancer increase the invasiveness of cancer cells (Chen et al., 2011). TAMs secrete PD-L1 to inhibit cytotoxic T cells and IL-10 to activate Treg (Zhu et al., 2016; Fang et al., 2021). Additionally, when PD-L1 is inhibited, TAMs may retain tumor immunosuppressive potential by boosting PD-L2 secretion (Umezu et al., 2019). TAMs can directly attract Treg cells to the site of the immunosuppressive milieu by generating CCL20 and CCL22, and they can also activate them by secreting IL-10 and TGF-β (Curiel et al., 2004; Biswas and Mantovani, 2010; Umezu et al., 2019). IL-6 and IL-10 are a group of cytokines, which are inducing tumor invasion and angiogenesis (Tamura et al., 2018). Recent studies showed that TAMs decrease E-cadherin by activating the TLR4/IL-10 signaling pathway promoting epithelial-to-mesenchymal transition in pancreatic cancer (Liu et al., 2013; Yao et al., 2018). Additionally, in transgenic mice models, IL-10 produced by TAMs is the key mediator in tumor resistance to paclitaxel and carboplatin (Ruffell et al., 2014). The IL-6 produced by TAMs promotes chemotherapy resistance in cancer cells by inhibiting the expression of miR-204-5p and activating the STAT3 pathway (Zhu et al., 2017).

### 4.3. MicroRNA in macrophage exosomes as critical regulators of the tumor microenvironment

**Figure 3:**
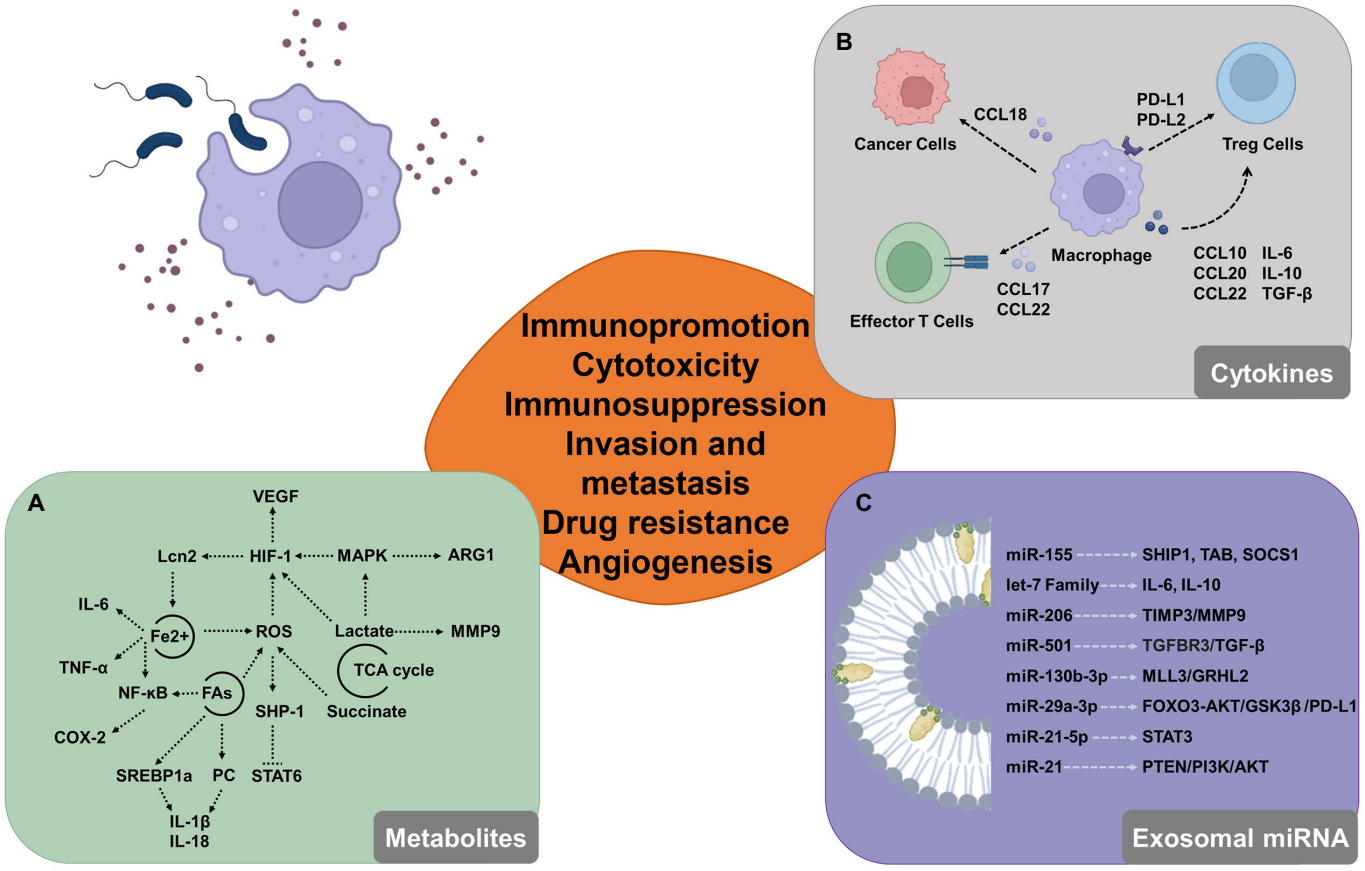
Potential involvement for macrophages that have been triggered by the microbiome in the tumor microenvironment. **(A)** By modifying the expression of intermediate metabolites, activated macrophages can modify glucose metabolism, iron cycling, and lipid metabolism and control the tumor microenvironment. **(B)** Activated macrophages release a variety of cytokines, chemokines, and growth factors that influence tumor formation by boosting cell proliferation and decreasing the activity of immune cells that can destroy tumors, like cytotoxic T cells. **(C)** Macrophage-derived exosomal miRNAs alter the immunological milieu by targeting proteins and activating molecules.

Several proteins, including SHIP1, TAB2, and SOCS1, in the innate immune signaling pathways, are targeted by the miR-155 gene, which is increased in macrophages after LPS infection and alters affects the expression of inflammatory mediators (Androulidaki et al., 2009; Ceppi et al., 2009; Cremer et al., 2009). Through a post-transcriptional break, *Salmonella* can cause the let-7 family to suppress the expression of IL-6 and IL-10 (Schulte et al., 2011). In THP-1 cells infected with *M. tuberculosis*, miR-206 expression is noticeably elevated, and this raised miR-206 favorably regulates inflammatory cytokines and MMP9 *via* targeting TIMP3 (Fu et al., 2016). These are shown miRNA could regulate the modulation of innate immunity signaling pathways. The primary mechanism for extracellular miRNA synthesis uses exosomes with energy-dependent active secretion. Exosomes are extremely small extracellular vesicles that contain proteins, lipids and nucleic acids, among other active components (Tan et al., 2020). In pancreatic ductal adenocarcinoma, it has been discovered that TAM-EVs transport miR-501-3p to suppress TGFBR3 expression, activate the TGF-β pathway, and encourage tumor migration and invasion (Yin et al., 2019). The advancement of gastric cancer is aided by M2 macrophage-derived extracellular vesicles through a miR-130b-3p/MLL3/GRHL2 signaling cascade (Zhang et al., 2020b). Exosomal miRNAs may have an impact on the biology of different cell types in TME. The STAT3 pathway is inhibited and Treg/Th17 cell imbalance is produced by TAM-EVs enriched in miR-21-5p and miR-29a-3p (Zhou et al., 2018). MiR-29a-3p controlled the FOXO3/AKT/GSK3 axis to suppress the expression of PD-L1, and PD-L1 expression can impair CD8 + T cell activity, causing immunological escape (Lu et al., 2021). TAM-EVs also contribute significantly to the pathophysiology of tumor chemoresistance (Figure 3). MiR-21 from tumor-associated macrophages that is transferred exosomal provides cisplatin resistance on gastric cancer cells by enhanced activation of PI3K/AKT signaling pathway by down-regulation of PTEN (Zheng P. et al., 2017).

## 5. Discussion

Studies on the microbiome have progressed from focusing on the cultivation of oral and intestinal bacteria to mechanistically understanding the link between host and microbiome, and more recently, to microbial profiles of all ecological niches in the body. The use of specific microbial signatures of cancer types may improve early and minimally invasive diagnostic approaches, and in this review, we discuss the bacteria in different cancer. However, the part of cancer most clearly associated with bacterial species remains ill-defined. The speculation of the presence of microbiota within tumors was finally confirmed by sequencing-based diagnosis, but there are many challenges in discriminating tumor-specific bacteria. The most frequent issues are biased results brought on by the use of various tissue sample processing techniques and the confounding of chemicals or environmental pollutants. Exogenous bacteria can now be used as potential immunotherapeutic agents or as a neoadjuvant in the treatment of cancer (St Jean et al., 2008). We will be able to identify distinct pathways that can be exploited for diagnostic, preventative, and therapeutic purposes if we can more precisely identify a particular strain in the tumor.

The host’s metabolism and immunity can be altered by the microbiota and its secreted components, which in turn can affect antitumor immunity. Numerous pathways suggest that bacteria acting as foreign microorganisms may indirectly contribute to the beginning or development of cancer. Unexpectedly, bacteria may multiply in macrophages and control them *via* a variety of interference tactics (Rosenberger and Finlay, 2003). During tumorigenesis and regression, macrophage polarization appears to act as an intermediate process that is activated by certain signals. Macrophages exhibit different phenotypes after receiving multiple stimulations which act on different receptors and thus exert regulatory effects by acting on multiple signaling pathways. It is a complicated story about how bacteria and macrophages interact. It should be emphasized that host cells are frequently cultivated *in vitro* for the majority of research against pathogens, including bacteria. Macrophage polarization is a result, and the molecular pathways of bacteria-driven macrophage polarization ought to connect to a particular environment concurrently, as the tumor microenvironment is a special environment that has the potential to preferentially generate macrophage polarization. In this article, we went over the molecular mechanisms of bacterial-induced macrophage polarization as well as the impact of activated macrophages on the tumor microenvironment.

A causal link between microbial enrichment in cancer and cancer itself cannot be shown, but understanding the relationships between microbes, macrophages, and cancer cells requires in-depth functional analysis per microbial scale is necessary. On one hand, one way to increase the efficiency and safety of tumor-targeted medicines is to modify bacteria using research on such pathogen-macrophage interactions. On the other hand, molecular mechanisms of cell polarization provide information and guidance for switching macrophage polarity in cancer.

## Author contributions

SX, YX, BF, DG, ZS, XL, and HW contributed to the study’s conception and design and commented on previous versions of the manuscript. Material preparation, data collection, and analysis were performed by BF, DG, and ZS. The first draft of the manuscript was written by SX and YX. All authors contributed to the article and approved the submitted version.

## Funding

This work was supported by National Natural Science Foundation of China (No. 81970008 and 82000020), Fundamental Research Funds for the Central Universities (2019CDYGZD009 and 2020CDJYGRH-1005), Natural Science Foundation of Chongqing, China (cstc2020jcyj-bshX0105 and cstc2020jcyj-msxmX0460) and Chongqing Talents: Exceptional Young Talents Project (No. cstc2021ycjh-bgzxm0099). The funders had no role in study design, data collection and analysis, decision to publish, or preparation of the manuscript.

## Conflict of interest

The authors declare that the research was conducted in the absence of any commercial or financial relationships that could be construed as a potential conflict of interest.

## Publisher’s note

All claims expressed in this article are solely those of the authors and do not necessarily represent those of their affiliated organizations, or those of the publisher, the editors and the reviewers. Any product that may be evaluated in this article, or claim that may be made by its manufacturer, is not guaranteed or endorsed by the publisher.

## Supplementary material

The Supplementary material for this article can be found online at: https://www.frontiersin.org/articles/10.3389/fmicb.2023.1115556/full#supplementary-material

## Figures and Tables

**Table:** 

TAMs	Tumor-associated macrophages
ROS	Reactive oxygen species
TNF-α	Tumor necrosis factor-α
IL-	Interleukin-
CXCL	C-X-C motif chemokine ligand
CCL	C-C motif chemokine ligand
TGF-β	Transforming growth factor β
TLRs	Toll-like receptors
STAT3	Signal transducer and activator of transcription 3
c-MYC	Cellular myelocytomatosis viral oncogene
NF-κB	Nuclear factor kappa-B
MRP14/S100A9	Myeloid-related protein-14
MyD88	Myeloid differentiation factor 88
AI-2	Autoinducer-2
TNFSF9	TNF receptor superfamily member 9
SCARI1/CD163	Scavenger receptor cysteine-rich type 1 protein M130
PRRs	Pattern recognition receptors
NLRs	NOD-like Receptors
MMPs	Matrix metalloproteinases
MHC	Major histocompatibility complex
miRNAs	micro-RNAs
CIITA	Major histocompatibility complex (MHC) class II transactivator
HLAII	Human leukocyte antigen (HLA) class II
PI3K	Phosphatidylinositide 3-kinases
AKT/PKB	Protein kinase B
LPS	Lipopolysaccharides
iNOS	Inducible nitric oxide synthase
HDAC	Histone deacetylase
HLA-DR	Human leukocyte antigen DR
MR/CD206	Mannose receptor
TCA cycle	Tricarboxylic acid cycle
VEGF	Vascular endothelial growth factor
ARG-1	Arginase 1
HIF-1α	Hypoxia-inducible factor-1α
MAPK	Mitogen-activated protein kinase
Lcn-2	Lipocalin 2
SOX9	Sex-determining region of the Y chromosome (SRY)-box transcription factor 9
COX-2	Cyclooxygenase-2
SREBP-1a	Sterol regulatory element binding proteins transcription factor 1a
SHP-1/PTPN6	Protein tyrosine phosphatase
Th	T helper cell
Tregs	Regulatory T cells
PITPNM3	PITPNM family member 3
PD-L1	Programmed death-ligand 1
PD-L2	Programmed death-ligand 2
SHIP1	Src homology 2‑containing inositol‑5′‑phosphatase 1
TAB2	TGF-β activated kinase 1 (MAP3K7) binding protein 2
SOCS1	Suppressors of cytokine signaling 1
TIMP3	TIMP Metallopeptidase Inhibitor 3
TGFBR3	Transforming growth factor beta receptor 3
MLL3	Myeloid/lymphoid or mixed lineage leukemia 3
GRHL2	Grainy head-like transcription factor 2
FOXO3	Forkhead box O3
GSK-3β	Glycogen synthase kinase-3β
MMAC1/PTEN	Mutated in multiple advanced cancers 1
