# Supplementary material for: Bacteria and macrophages in the tumor microenvironment
Source: Front Microbiol. 2023 Feb 7;14:1115556. doi: 10.3389/fmicb.2023.1115556 (PMC9941202; doi:10.3389/fmicb.2023.1115556)
Supplement: Supplementary file 1 [file Table_1.DOCX]

**Table 1.** Summary of the cancer microbiome. The species of bacteria have been reported in studies that present in cancer.

| Cancer | Phylum | Genus | references |
| --- | --- | --- | --- |
| Colorectal Cancer | *Bacteroidetes* | *Bacteroides* | (1, 10-14) |
| Colorectal Cancer | *Bacteroidetes* | *Prevotella* | (11-13, 15) |
| Colorectal Cancer | *Bacteroidetes* | *Porphyromonas* | (14-16) |
| Colorectal Cancer | *Firmicutes* | *Peptostreptococcus;*  *Solobacterium* | (13, 15, 16) |
| Colorectal Cancer | *Firmicutes* | *Streptococcus* | (13, 14) |
| Colorectal Cancer | *Firmicutes* | *Clostridium;*  *Gemella* | (13, 15) |
| Colorectal Cancer | *Firmicutes* | *Lachnospiraceae* | (12, 14) |
| Colorectal Cancer | *Firmicutes* | *Roseburia* | (17) |
| Colorectal Cancer | *Proteobacteria* | *Escherichia* | (12, 16, 18-20) |
| Colorectal Cancer | *Proteobacteria* | *Campylobacter* | (21) |
| Colorectal Cancer | *Actinobacteria* | *Bifidobacterium* | (22) |
| Colorectal Cancer | *Actinobacteria* | *Parvimonas* | (13-16) |
| Colorectal Cancer | *Fusobacteria* | *Fusobacterium* | (10-16, 23-32) |
| Stomach Cancer | *Bacteroidetes* | *Alloprevotella* | (33, 34) |
| Stomach Cancer | *Firmicutes* | *Parvimonas* | (33-38) |
| Stomach Cancer | *Firmicutes* | *Dialister* | (33-36, 39) |
| Stomach Cancer | *Firmicutes* | *Streptococcus* | (35, 36, 40-42) |
| Stomach Cancer | *Firmicutes* | *Slackia* | (33-36, 43, 44) |
| Stomach Cancer | *Firmicutes* | *Lactobacillus* | (33, 40) |
| Stomach Cancer | *Firmicutes* | *Clostridium* | (40, 45) |
| Stomach Cancer | *Firmicutes* | *Staphylococcus* | (34, 46) |
| Stomach Cancer | *Firmicutes* | *Veillonella* | (47) |
| Stomach Cancer | *Proteobacteria* | *Helicobacter* | (40, 48) |
| Stomach Cancer | *Proteobacteria* | *Neisseria* | (33, 41) |
| Stomach Cancer | *Proteobacteria* | *Sphingobium* | (47) |
| Stomach Cancer | *Proteobacteria* | *Escherichia;*  *Burkholderia* | (41) |
| Stomach Cancer | *Fusobacteria* | *Fusobacterium* | (40) |
| Esophageal Cancer | *Firmicutes* | *Lactobacillus;*  *Streptococcus* | (49) |
| Esophageal Cancer | *Fusobacteria* | *Fusobacterium* | (50) |
| Pancreatic Cancer | *Bacteroidetes* | *Porphyromonas* | (51-54) |
| Pancreatic Cancer | *Firmicutes* | *Streptococcus;*  *Granulicatella* | (55) |
| Pancreatic Cancer | *Proteobacteria* | *Pseudoxanthomonas* | (52) |
| Pancreatic Cancer | *Proteobacteria* | *Neisseria* | (55) |
| Pancreatic Cancer | *Actinobacteria* | *Saccharopolyspora;*  *Streptomyces* | (52, 54) |
| Gallbladder Cancer | *Bacteroidetes* | *Bacteroidaceae;*  *Prevotellaceae;*  *Porphyromonadaceae* | (56) |
| Gallbladder Cancer | *Firmicutes* | *Veillonellaceae* | (56) |
| Gallbladder Cancer | *Proteobacteria* | *Salmonella* | (57-60) |
| Gallbladder Cancer | *Proteobacteria* | *Helicobacter* | (61-63) |
| Gallbladder Cancer | *Proteobacteria* | *Escherichia* | (64) |
| Gallbladder Cancer | *Proteobacteria* | *Enterobacteriaceae* | (64) |
| Gallbladder Cancer | *Fusobacteria* | *Fusobacterium* | (64) |
| Lung Cancer | *Bacteroidetes* | *Prevotella* | (65-67) |
| Lung Cancer | *Bacteroidetes* | *Capnocytophaga* | (68, 69) |
| Lung Cancer | *Firmicutes* | *Streptococcus* | (65-68, 70-73) |
| Lung Cancer | *Firmicutes* | *Veillonella* | (65-67, 69, 74) |
| Lung Cancer | *Firmicutes* | *Staphylococcus* | (66, 71) |
| Lung Cancer | *Firmicutes* | *Lactobacillus* | (67, 75) |
| Lung Cancer | *Firmicutes* | *Gemella* | (67) |
| Lung Cancer | *Firmicutes* | *Selenomonas* | (69) |
| Lung Cancer | *Firmicutes* | *Enterococcus* | (73) |
| Lung Cancer | *Firmicutes* | *Megasphaera* | (74) |
| Lung Cancer | *Proteobacteria* | *Enterobacter* | (7, 8, 71, 73) |
| Lung Cancer | *Proteobacteria* | *Acinetobacter* | (8, 73) |
| Lung Cancer | *Proteobacteria* | *Haemophilus* | (67, 71) |
| Lung Cancer | *Proteobacteria* | *Burkholderia* | (66, 67) |
| Lung Cancer | *Proteobacteria* | *Moraxella* | (67) |
| Lung Cancer | *Proteobacteria* | *Neisseria* | (69) |
| Lung Cancer | *Proteobacteria* | *Noviherbaspirillum;*  *Aggregatibacter* | (75) |
| Lung Cancer | *Proteobacteria* | *Brevundimonas* | (7, 8) |
| Lung Cancer | *Proteobacteria* | *Acidovorax* | (76) |
| Lung Cancer | *Proteobacteria* | *Morganella;*  *Escherichia* | (77) |
| Lung Cancer | *Proteobacteria* | *Legionella* | (78) |
| Lung Cancer | *Actinobacteria* | *Rothia* | (65, 67) |
| Lung Cancer | *Actinobacteria* | *Propionibacterium* | (7, 8) |
| Lung Cancer | *Fusobacteria* | *Fusobacterium* | (67) |
| Lung Cancer | *Deinococcus-Thermus* | *Thermus* | (78) |
| Lung Cancer | *Verrucomicrobia* | *Akkermansia* | (79, 80) |
| Breast Cancer | *Firmicutes* | *Bacillus;*  *Staphylococcus* | (81) |
| Breast Cancer | *Proteobacteria* | *Enterococcus* | (81) |
| Breast Cancer | *Fusobacteria* | *Fusobacterium* | (82) |
| Cervical Cancer | *Bacteroidetes* | *Prevotella* | (83, 84) |
| Cervical Cancer | *Firmicutes* | *Lactobacillus* | (51, 85) |
| Cervical Cancer | *Firmicutes* | *Dialister;*  *Finegoldia Magna;*  *Peptoniphilus* | (83) |
| Cervical Cancer | *Firmicutes* | *Parvimonas;*  *Peptostreptococcus;*  *Anaerococcus* | (84) |
| Cervical Cancer | *Firmicutes* | *Clostridium* | (86) |
| Cervical Cancer | *Firmicutes* | *Streptococcus* | (86, 87) |
| Cervical Cancer | *Firmicutes* | *Megasphaera* | (84, 88) |
| Cervical Cancer | *Proteobacteria* | *Hydrogenophilus;*  *Burkholderia* | (89) |
| Cervical Cancer | *Actinobacteria* | *Atopobium* | (83, 84, 88, 90) |
| Cervical Cancer | *Actinobacteria* | *Gardnerella* | (83-85, 89) |
| Cervical Cancer | *Actinobacteria* | *Eggerthella* | (88) |
| Cervical Cancer | *Actinobacteria* | *Bifidobacterium* | (89) |
| Cervical Cancer | *Fusobacteria* | *Sneathia* | (84, 89-95) |
| Cervical Cancer | *Fusobacteria* | *leptotrichia* | (88) |
| Cervical Cancer | *Fusobacteria* | *Fusobacterium* | (89) |
| Prostate Cancer | *Bacteroidetes* | *Bacteroides* | (96-99) |
| Prostate Cancer | *Firmicutes* | *Staphylococcus* | (6, 100) |
| Prostate Cancer | *Firmicutes* | *Streptococcus* | (6, 98) |
| Prostate Cancer | *Firmicutes* | *Faecalibacterium* | (101, 102) |
| Prostate Cancer | *Firmicutes* | *Clostridium* | (103) |
| Prostate Cancer | *Proteobacteria* | *Escherichia* | (96, 104) |
| Prostate Cancer | *Proteobacteria* | *Proteus;*  *Aeromonas* | (104) |
| Prostate Cancer | *Proteobacteria* | *Campylobacter* | (105) |
| Prostate Cancer | *Actinobacteria* | *Propionibacterium* | (9, 100, 106) |
| Prostate Cancer | *Actinobacteria* | *Corynebacterium* | (6, 107) |
| Prostate Cancer | *Verrucomicrobia* | *Akkermansiaceae* | (107) |
