# Supplementary material for: Bacteria and macrophages in the tumor microenvironment
Source: Front Microbiol. 2023 Feb 7;14:1115556. doi: 10.3389/fmicb.2023.1115556 (PMC9941202; doi:10.3389/fmicb.2023.1115556)
Supplement: Supplementary file 2 [file Table_2.DOCX]

| **Abbreviations** |  |
| --- | --- |
| TAMs | Tumor-associated macrophages |
| ROS | Reactive oxygen species |
| TNF-α | Tumor necrosis factor-α |
| IL- | Interleukin- |
| CXCL | C-X-C motif chemokine ligand |
| CCL | C-C motif chemokine ligand |
| TGF-β | Transforming growth factor β |
| TLRs | Toll-like receptors |
| STAT3 | Signal transducer and activator of transcription 3 |
| c-MYC | Cellular myelocytomatosis viral oncogene |
| NF-κB | Nuclear factor kappa-B |
| MRP14/S100A9 | Myeloid-related protein-14 |
| MyD88 | Myeloid differentiation factor 88 |
| AI-2 | Autoinducer-2 |
| TNFSF9 | TNF receptor superfamily member 9 |
| SCARI1/CD163 | Scavenger receptor cysteine-rich type 1 protein M130 |
| PRRs | Pattern recognition receptors |
| NLRs | NOD-like Receptors |
| MMPs | Matrix metalloproteinases |
| MHC | Major histocompatibility complex |
| miRNAs | micro-RNAs |
| CIITA | Major histocompatibility complex (MHC) class II transactivator |
| HLAⅡ | Human leukocyte antigen (HLA) class II |
| PI3K | Phosphatidylinositide 3-kinases |
| AKT/PKB | Protein kinase B |
| LPS | Lipopolysaccharides |
| iNOS | **Inducible nitric oxide synthase** |
| HDAC | Histone deacetylase |
| HLA-DR | Human leukocyte antigen DR |
| MR/CD206 | Mannose receptor |
| TCA cycle | Tricarboxylic acid cycle |
| VEGF | Vascular endothelial growth factor |
| ARG-1 | Arginase 1 |
| HIF-1α | Hypoxia-inducible factor-1α |
| MAPK | Mitogen-activated protein kinase |
| Lcn-2 | Lipocalin **2** |
| SOX9 | Sex-determining region of Y chromosome (SRY)-box transcription factor 9 |
| COX-2 | Cyclooxygenase-2 |
| SREBP-1a | Sterol regulatory element binding proteins transcription factor 1a |
| SHP-1/PTPN6 | Protein tyrosine phosphatase |
| Th | T helper cell |
| Tregs | **Regulatory T cells** |
| PITPNM3 | PITPNM family member 3 |
| PD-L1 | Programmed death-ligand 1 |
| PD-L2 | Programmed death-ligand 2 |
| SHIP1 | Src homology 2‑containing inositol‑5'‑phosphatase 1 |
| TAB2 | TGF-β activated kinase 1 (MAP3K7) binding protein 2 |
| SOCS1 | Suppressors of cytokine signaling 1 |
| TIMP3 | TIMP Metallopeptidase Inhibitor 3 |
| TGFBR3 | Transforming growth factor beta receptor 3 |
| MLL3 | Myeloid/lymphoid or mixed lineage leukemia 3 |
| GRHL2 | Grainy head-like transcription factor 2 |
| FOXO3 | Forkhead box O3 |
| GSK-3β | Glycogen synthase kinase-3β |
| MMAC1/PTEN | Mutated in multiple advanced cancers 1 |
